# A randomised controlled trial of a tele-based lifestyle intervention for colorectal cancer survivors ('CanChange'): study protocol

**DOI:** 10.1186/1471-2407-9-286

**Published:** 2009-08-18

**Authors:** Anna L Hawkes, Kenneth I Pakenham, Kerry S Courneya, Sara Gollschewski, Peter Baade, Louisa G Gordon, Brigid M Lynch, Joanne F Aitken, Suzanne K Chambers

**Affiliations:** 1 Viertel Centre for Research in Cancer Control, Cancer Council Queensland, Brisbane, Australia; 2 School of Public Health, Queensland University of Technology, Brisbane, Australia; 3 School of Psychology, The University of Queensland, Brisbane, Australia; 4 Faculty of Physical Education and Recreation, University of Alberta, Edmonton, Canada; 5 Queensland Institute of Medical Research, Brisbane, Australia; 6 Cancer Prevention Research Centre, The University of Queensland, Brisbane, Australia; 7 Griffith Institute for Health and Medical Research, Griffith University, Brisbane, Australia

## Abstract

**Background:**

Colorectal cancer survivors may suffer from a range of ongoing psychosocial and physical problems that negatively impact on quality of life. This paper presents the study protocol for a novel telephone-delivered intervention to improve lifestyle factors and health outcomes for colorectal cancer survivors.

**Methods/Design:**

Approximately 350 recently diagnosed colorectal cancer survivors will be recruited through the Queensland Cancer Registry and randomised to the intervention or control condition. The intervention focuses on symptom management, lifestyle and psychosocial support to assist participants to make improvements in lifestyle factors (physical activity, healthy diet, weight management, and smoking cessation) and health outcomes. Participants will receive up to 11 telephone-delivered sessions over a 6 month period from a qualified health professional or 'health coach'. Data collection will occur at baseline (Time 1), post-intervention or six months follow-up (Time 2), and at 12 months follow-up for longer term effects (Time 3). Primary outcome measures will include physical activity, cancer-related fatigue and quality of life. A cost-effective analysis of the costs and outcomes for survivors in the intervention and control conditions will be conducted from the perspective of health care costs to the government.

**Discussion:**

The study will provide valuable information about an innovative intervention to improve lifestyle factors and health outcomes for colorectal cancer survivors.

**Trial Registration:**

ACTRN12608000399392

## Background

Colorectal cancer is the second most common cancer worldwide [1] with approximately 60% of people diagnosed surviving the disease [2]. Cancer survivors report a range of psychosocial and physical problems including: depression and anxiety, lowered self-esteem and body image; treatment side effects such as fatigue, pain, and nausea; reduced cardiovascular function; and muscle weakness [3,4]. An increasing incidence in cancer due to an ageing population coupled with a growing incidence of some cancers and improvements in early detection and treatment has seen an increase in the number of cancer survivors [5]. As such, there is increasing interest in how to ameliorate the ongoing problems for this growing population to ultimately enhance quality of life [6,7].

It is estimated that up to 40% of colorectal cancer survivors experience heightened psychological distress [8], and a significant proportion are not meeting current public health guidelines for lifestyle factors or health behaviours [9,10]. In the first 12 months following diagnosis, approximately 60% of colorectal cancer survivors are overweight, 70% are insufficiently active, and 22% are high risk drinkers [11]. Research has also shown that compared with non-distressed survivors, the distressed group have an increased likelihood of poor lifestyle factors including smoking, physical inactivity and obesity [12]. Therefore, it is clearly important to help survivors manage the adverse psychosocial and physical consequences of colorectal cancer and its treatment.

The period after cancer diagnosis and treatment has been described as a 'teachable moment". Despite the challenges of cancer diagnoses and treatment, cancer survivors have been found to be highly motivated to make behavioural improvements to optimise their health and prevent cancer recurrence [13-15]. Also, a growing body of research suggests that the chronic and late effects of cancer treatment are amenable to the same types of behavioural or lifestyle interventions that are applied to other chronic disease populations [16]. There have been several randomised controlled trials (RCT) of lifestyle interventions for cancer survivors that have resulted in improvements in a range of health outcomes, although they have predominately included breast cancer survivors [17]. Compared with breast cancer survivors, colorectal cancer patients present with more advanced disease, they undergo different medical and surgical procedures, they tend to be older and they include equal numbers of men and women. As such, the results from previous studies cannot be easily generalised to colorectal cancer survivors [18-20].

There have only been a few trials to address the specific needs of colorectal cancer survivors, including one RCT of a psychosocial intervention [21] and one RCT of a lifestyle intervention [18]. These studies have demonstrated effectiveness although both identified a number of limitations. The psychosocial trial included n = 249 survivors and the intervention group (n = 125) received 10 home-visits over two years from health professionals. The intervention group had significantly lower occurrence of symptoms of fatigue at three months follow-up, however there were no intervention effects at two years. This study was limited by differential compliance with the follow-up interviews, and the control group received some psychosocial intervention due to inexperienced staff. Furthermore, the content of the home visits was not theory-driven or structured as the topics discussed were set by the participants [21]. The lifestyle trial [18] included 102 survivors and the intervention group (n = 69) completed a home-based, personalised exercise program for 20–30 minutes three to five times per week. Intention-to-treat analyses revealed no differences in quality of life between the intervention and control groups, although exploratory analyses found that intervention participants who increased their physical fitness reported higher levels of quality of life and lower levels of anxiety and depression. Investigators reported exercise contamination in the control group as the study team contacted both the intervention and control participants weekly to report their exercise levels.

Previous research has suggested that interventions to improve quality of life after colorectal cancer may be most effective if they target symptom management, psychosocial and lifestyle variables, or health behaviours, in a comprehensive and integrated approach [22]. Further, patients have expressed the need for support with symptom management, psychosocial and lifestyle factors following a diagnosis of colorectal cancer [23]. However, despite the importance of comprehensive support and the promising results of previous interventions, to our knowledge there has been no previous trial of a broad and integrated supportive care program for colorectal cancer survivors. Accordingly, we have designed an intervention that focuses on symptom management, psychosocial and lifestyle support for recently diagnosed colorectal cancer survivors.

The intervention includes an evidenced-based approach with strategies drawn from the core components of Acceptance and Commitment Therapy (ACT) [24-27]. ACT is an empirically based third generation cognitive behavioural approach that uses acceptance and mindfulness strategies, and commitment and behaviour change strategies to produce psychological flexibility: the ability to defuse from difficult thoughts and accept difficult feelings while persisting in values-based action [24-28] Psychological flexibility is established through six core ACT processes: acceptance, cognitive defusion (changing our relationship with thoughts), being present, self-as-context, values and committed action [26]. The approach explicitly teaches strategies designed to increase tolerance in the service of goal-directed behaviour, such as healthy eating and physical activity.

Importantly, we are not trialling a psychotherapeutic intervention; rather ACT strategies are used to enhance positive lifestyle behaviours. We propose that ACT provides an alternative to existing intervention approaches for cancer survivors, using specific strategies to overcome internal barriers to making lifestyle improvements by emphasizing the role of emotions and thoughts in the maintenance of good self-management of lifestyle factors. To date, ACT interventions have been successfully used to enhance quality of life and promote positive lifestyle behaviours for a range of health conditions including diabetes [29,30], chronic pain [31], smoking [32], epilepsy [33], and obesity or weight management [34-38].

CanChange will overcome a number of the study limitations of previous trials by trialling a comprehensive and integrated intervention that is theory-based, structured and promotes psychological flexibility to enhance behaviour change. As well, the intervention will be delivered by highly skilled health professionals over the telephone. Tele-based support has been shown to be highly acceptable for people diagnosed with cancer [23,39,40], can overcome geographical barriers to access, and can overcome many of the reported barriers to participation in facility-based support programs (e.g. transport, return to work, illness). In addition, telephone-delivered interventions are convenient and flexible; they can be delivered at a suitable time for the participant and in their own home; and importantly they improve behavioural outcomes for cancer survivors [40]. Finally, telephone interventions may be immediately translatable into broad-reach telephone helplines that are used widely for cancer patients in Australia and other countries. Whilst telephone interventions cannot reach those without access to a telephone, in Australia, approximately 96% of the population live in a household with at least one telephone connection [41].

This paper presents the protocol for an RCT to evaluate the effectiveness of an intervention to improve lifestyle factors and health outcomes for colorectal cancer survivors. We will also investigate the cost-effectiveness of the intervention. We hypothesise that compared with participants receiving the control condition, intervention participants will have improved lifestyle factors and health outcomes. Additionally, we hypothesise that the intervention will be cost-effective compared with the control condition. The results of this study will provide valuable new information about an intervention to improve lifestyle behaviours and health outcomes for colorectal cancer survivors.

## Methods/Design

### Study Design

The study is a two-armed prospective RCT in which approximately 350 colorectal cancer survivors will be randomised in a 1:1 ratio to the intervention or control group using a computer-generated random numbers sequence. Participants in both groups will complete assessments at baseline (Time 1), post-intervention or 6 months follow-up (Time 2), and at 12 months follow-up for longer term effects (Time 3).

### Study Aims

i. To investigate the effect of a lifestyle intervention on lifestyle factors and health outcomes [primary outcome variables include physical activity, cancer-related fatigue and quality of life] at Time 2 and Time 3.

ii. To assess the cost-effectiveness of study participants receiving the intervention in comparison with participants receiving the control condition.

### Sample Recruitment Procedures

Ethics approval was received from Human Research Ethics Committees of the University of Queensland's Behavioural and Social Sciences Ethical Review Committee (approval number 2007000656) and from 18 private and public hospitals throughout Queensland. Eligibility criteria include: (i) adult persons resident in Queensland; (ii) a histologically confirmed diagnosis of primary colorectal cancer (C18-C20, C218) within the previous 12 months and notified to the Queensland Cancer Registry over a nine month period (October 1^st ^2008 to June 30^th ^2009); (iii) ability to understand and provide written informed consent in English; (iv) no metastatic disease; (v) no medical conditions that would limit adherence to an unsupervised lifestyle program (as confirmed by their referring physician); (vi) a telephone; and (vii) at least one poor lifestyle factor [routinely exercise <150 minutes per week [42], do not adhere to a healthy diet (indicated by < 2 serves fruit and 5 serves vegetables per day [43], *or *overweight (body mass index, BMI>=25) [44].

Based on Queensland incidence figures and available evidence on the proportion of Queensland colorectal cancer survivors with poor lifestyle behaviours (insufficiently active, unhealthy diet or overweight) [11], we expect n = 875 potential participants over a nine month recruitment period. Patients' names and their doctors will be obtained from the Queensland Cancer Registry. As is required Registry procedure, initial letters will be sent to each patient's doctor requesting doctor's permission to approach the patient, followed by reminder telephone calls. With doctor's permission, patients will be sent the consent package and interested patients will provide written informed consent in the presence of a witness.

Based on our past experience of recruiting colorectal cancer patients with this method, we anticipate approximately 80% of doctors will give permission for patients to be contacted and approximately 60% of patients will consent to participate. We therefore anticipate a final sample size of approximately n = 350 eligible participants (n = 175 in the intervention and control conditions at Time 1). Conservatively allowing for 20% attrition during the intervention, we expect n = 140 subjects per condition at Time 2. Sample size analysis indicated that 130 subjects per condition (intervention and control) or a total of 260 were required to detect, with 80% power and type I error of 5% (two-tailed), a moderate effect size in quality of life of 0.35.

Upon receipt of the completed consent package, all patients will be phoned by the project team to complete a brief screening instrument to assess factors that would exclude participation in the trial (physical activity using the Godin Leisure-Time Exercise Questionnaire [45], fruit and vegetable intake using brief questions developed by the Cancer Council NSW [46], and BMI elicited from self reported weight and height. Telephone interviewers will then complete the Time 1 interview, and computer-generated randomisation to study condition will occur following the completion of the Time 1 interview. Randomisation will be conducted by the project manager and concealed from investigators.

### Study Conditions

#### Control

Control participants will receive existing educational brochures produced by Cancer Council Australia on bowel cancer and lifestyle factors. During the study period, they will receive a quarterly study newsletter to enhance participant retention, and will be contacted for all follow-up assessments.

#### Intervention

The intervention will include (i) telephone delivered health coaching sessions; (ii) participant handbook, (iii) regular motivational postcard prompts; (iv) pedometer; (v) and the quarterly study newsletter sent to control participants. The intervention will be detailed in an intervention manual including health coaching session scripts and worksheets. The intervention will focus on symptom management, as well as psychosocial and lifestyle support to help participants manage the current and possible future stresses associated with colorectal cancer and make positive lifestyle changes. The intervention framework is based on ACT as outlined previously [24,26] and limited session content has been drawn from a current trial of a psychosocial and physical activity intervention to improve heart health [47].

The sessions will include: the cancer experience; colorectal cancer-related symptoms; specific ACT components in relation to lifestyle behaviours (values, mindfulness, defusion or mindfulness of thoughts, acceptance or mindfulness of feelings, and committed action); and strategies to enhance improvement in lifestyle behaviours consistent with the national recommendations and individual goals [42-44,48-51]. Intervention strategies will be consistent with the session content including: ACT-strategies; motivational interviewing; problem solving; action planning and goal setting; as well as reviewing and ongoing monitoring.

The health coaching sessions will be delivered by study-trained health coaches who will be guided by a custom developed web-based computer application and will key enter all session information. Health coaches will receive comprehensive training in: colorectal cancer incidence, treatment, symptoms and outcomes; ACT, behavioural models of health and illness and behaviour change; as well as national guidelines for lifestyle behaviours [42-44,48-51]. Role-play and simulated calls will be conducted to prepare health coaches for program delivery. The first 10 sessions will be delivered bi-weekly over a five month period, followed by the final telephone session four weeks later to promote self management techniques and long term maintenance of lifestyle improvements.

The participant handbook will include educational information on lifestyle behaviours and the core components of ACT. The handbook will also include additional information sheets on common problems faced by colorectal cancer survivors such as managing the side effects of treatment, as well as tracking and monitoring tables for behaviour change.

Participants will receive motivational postcard prompts in the mail between telephone sessions based on the previous session and action plan developed by the study participant. These postcards will aim to promote behaviour change and participant retention. Participants will also be issued with a Yamax SW700 Multifunction Digi-Walker pedometer at Time 1 to assist with the promotion of physical activity throughout the intervention. Health coaches will explain how to use the pedometer and the handbook will include an informational sheet. Participants will be encouraged to achieve 10,000 steps a day, and to track and monitor their steps throughout the intervention.

### Study Integrity

The study design will be guided by the CONSORT statement [52], and randomisation to study condition will occur following the completion of Time 1 assessment. Project staff tracking data collection will be blinded to study condition. Randomisation will occur using block randomisation undertaken by the study manager and concealed from investigators. The intervention protocol will be manualised, and all intervention calls will be audio-taped with a proportion reviewed to ensure adherence to the delivery of the intervention protocol. All analyses will be conducted on the basis of intention to treat.

### Measurement

The majority of study data will be collected at Time 1, Time 2 and Time 3 by computer assisted telephone interview (CATI). Self-reported socio-demographic variables will include gender, age, ethnicity, income, education, employment status, and private health insurance status. Medical information will include co-morbidities and colorectal cancer treatment. Disease variables including cancer grade and stage, as available, will be collected from cancer registry records. To assess the implementation of the intervention, we will measure intervention satisfaction and quality of materials by self-administered questionnaire at Time 2, participant adherence to the intervention and level of contact by data collected from the web-based computer application, and delivery of the intervention components through a health coaching session checklist.

#### Implementation Evaluation

A self-administered satisfaction survey will be used to measure satisfaction with specific aspects of the intervention as well as overall satisfaction with the intervention. Specific questions will address: the content (e.g. relevance and usefulness of topics); the service (e.g. timing and duration of calls, use of the telephone); the relationship with the health coach (e.g. expectations met, feeling comfortable, level of rapport with the health coach); and the intervention materials (e.g. relevance and readability of the handbook and brochures). Participants will also be asked to highlight the strengths and weaknesses of the intervention in two open-ended questions.

The delivery of the intervention will be evaluated by an objective assessor using a criterion based checklist for each health coaching session. The criteria will include assessment of health coaching skills used (e.g rapport building) as well as a checklist against the content objectives for each session (e.g. how well did the coach explain the purpose of the call?). Participant adherence to the intervention and level of contact will be assessed by: the proportion of sessions completed during the intervention period; the topics covered in each session; the total length (minutes) of intervention exposure during the intervention period; and the number of call attempts, missed calls and reasons for missed calls. This data will be recorded by health coaches on a web-based computer application throughout the intervention.

#### Outcome Evaluation

##### Primary outcome variables

###### Physical activity

We will use a modified version of the leisure score index of the Godin Leisure-Time Exercise Questionnaire that has been shown to be a reliable and valid self-report measure of physical activity[45]. The questionnaire contains three questions that assess the average frequency of mild, moderate, and strenuous exercise during free time in a typical week. The modified version of the questionnaire also provides average duration.

###### Cancer-related fatigue

We will use the 13-item Functional Assessment of Chronic Illness Therapy Fatigue Scale (FACT-FS) of the Functional Assessment of Chronic Illness Therapy measurement system which was developed specifically for the cancer population[53,54].

###### Quality of life

We will use the Short Form-36 (SF-36), a widely used measure that has published norms for the Australian general population[55,56]. The SF-36 contains a mental health and physical health summary scale suitable to measure the impact of the intervention on patients' wellbeing, and generates a preference-based utility instrument SF-6D which will be used in the cost-effectiveness analyses.

##### Secondary Outcome Variables

###### Nutrition

We will use the Cancer Council Victoria Food Frequency Questionnaire that shows acceptable levels of reliability and validity when compared with seven-day weighted food records, and has been successfully used over the telephone with cancer survivors[57].

###### Smoking

We will use brief questions about smoking behaviour based on the commonly used Cancer Council New South Wales validated items[46].

###### Weight management

Will be assessed by body mass index (BMI, kg/m^2^) and waist circumference (cm). Participants will be provided with tape measures for measurement of waist circumference.

###### Psychological adjustment

We will use (a) the Brief Symptom Inventory (BSI-18) to assess anxiety, depression and somatisation[58]; (b) the Acceptance and Action Questionnaire (AAQ-II) to assess experiential avoidance and the Mindful Attention Awareness Scale (MAAS)[59] to assess mindfulness; and (c) The post-traumatic growth inventory (PTGI) to assess growth following a cancer diagnosis[60]; and (d) the Functional Assessment of Chronic Illness Therapy Spiritual Well-being module (FACIT-Sp)[61] to measure spirituality.

###### Colorectal cancer specific quality of life and physical symptoms

We will use the Functional Assessment of Chronic Illness Therapy colorectal cancer (FACT-C) to measure quality of life and colorectal cancer-specific symptoms [53,54].

#### Cost Effectiveness

In order to assess the economic value of the intervention, several health outcome variables (detailed above) will be combined with resource data to produce cost-effectiveness ratios at Time 2. Taking a 'health system' perspective, the types of resources in the analyses will include all intervention resources recorded by the study manager (e.g. health coach materials), participant health care utilisation (e.g. hospital admissions), community health resources and medications. Detailed data will be collected by self report and validated with a randomised sub-sample of 20% of patient records and provider surveys. Resources will be valued using nationally applicable sources (e.g. Diagnosis-Related Group codes for hospital admissions and Pharmaceutical Benefits Scheme cost codes for medications). All costs will be aggregated for the control and intervention arms. The primary health benefit for the cost effectiveness analyses will be quality-adjusted life years (QALYs). Quality of life will be measured by the SF-36; the SF6D algorithm will be used to produce QALY-weights on a 0–1 scale. Secondary health benefits will include the mean change in lifestyle factors and health outcomes.

### Data Analyses

Each hypothesis will be tested independently, taking into account hypothesised predictors as well as potential confounding factors (e.g. age, gender). Multiple (linear, multinomial or logistic) regression models will be fitted to consider the relative strength of cross-sectional associations between primary outcome variables (continuous, categorical or dichotomous) and patient characteristics. Repeated-measures regression models will be used to analyse and summarise changes in primary outcome variables over time and to determine the relationships with the various demographic, clinical, lifestyle, and psychosocial characteristics over the three measured time points. The effect of the intervention will be assessed by including a dichotomous variable (control/intervention) in the model, and assessing its interaction with the time-dependent response variable. Repeated-measures regression models will use a generalised estimating equations approach to account for the expected missing data due to attrition over time. Variables measuring health outcomes at Time 1 will be included in subsequent models to control for their effect on health outcomes in later time periods. Exploratory analyses on effect modification and mediation will also be conducted. Effect modification will be assessed through subgroup analyses and the introduction of interaction terms into the models to detect statistical differences between stratum-specific estimates. Standard statistical software including STATA (STATA SE 10.0; StataCorp, College Station, TX), and SPSS (version 14.0 for Windows) will be used for analysis and data management.

For the cost effectiveness analyses, incremental cost-effectiveness rations combining cost and health outcome data will be calculated. The incremental difference in costs between the 2 groups will be divided by the difference in QALY's (and other secondary health benefits) between the groups at Time 2 to generate the incremental cost-effectiveness ratio. This represents the additional cost and health effects of the intervention above the control condition. One-way sensitivity analyses will be undertaken to test the robustness of the ratios to variation in key parameters. Detailed probabilistic (multivariate) sensitivity analysis will be undertaken for all projections and parameters with uncertainty and/or variability using TreeAge Pro 2009 (Healthcare module) software (TreeAge Software Inc, Williamstown, MA, USA).

## Discussion

This study will trial a population based approach to promote lifestyle improvements and health outcomes for colorectal cancer survivors. For colorectal cancer survivors, no intervention studies to date have targeted the specific needs of colorectal cancer survivors in a comprehensive and integrated intervention focusing on symptom management, psychosocial and lifestyle support; or investigated the cost-effectiveness of this approach. If successful, the intervention will be immediately translatable into practice by trained staff in a range of settings including broad reach telephone help lines available internationally, or through acute health care settings.

## Competing interests

The authors declare that they have no competing interests.

## Authors' contributions

ALH, SC, JA, BML and KP developed the study concept and all authors further developed the study protocol. ALH and KP are responsible for the implementation of the intervention. ALH was responsible for drafting the manuscript and all authors contributed to the final manuscript.

## Pre-publication history

The pre-publication history for this paper can be accessed here:

http://www.biomedcentral.com/1471-2407/9/286/prepub
